# Dendropanoxide Alleviates Thioacetamide-induced Hepatic Fibrosis via Inhibition of ROS Production and Inflammation in BALB/_C_ Mice

**DOI:** 10.7150/ijbs.80743

**Published:** 2023-05-11

**Authors:** Amit Kundu, Sreevarsha Gali, Swati Sharma, Sam Kacew, Sungpil Yoon, Hye Gwang Jeong, Jong Hwan Kwak, Hyung Sik Kim

**Affiliations:** 1 School of Pharmacy, Sungkyunkwan University, 2066, Seobu-ro, Jangan-gu, Suwon 440-746, Republic of Korea.; 2 McLaughlin Centre for Population Health Risk Assessment, University of Ottawa, Ottawa, ON, Canada.; 3 College of Pharmacy, Chungnam National University, 99 Daehak-ro, Yuseong-Gu, Daejeon 34134, Republic of Korea.; 4 School of Medical Sciences, Örebro University, Örebro, Sweden; Cardiovascular Research Centre (CVRC), School of Medical Sciences, Örebro University, Örebro, Sweden.

**Keywords:** Hepatic fibrosis, Thioacetamide, Oxidative stress, Inflammation, Apoptosis, Dendropanoxide

## Abstract

Hepatic fibrosis results from overproduction and excessive accumulation of extracellular matrix (ECM) proteins in hepatocytes. Although the beneficial effects of dendropanoxide (DPx) isolated from *Dendropanax morbifera* have been studied, its role as an anti-fibrotic agent remains elucidated. We investigated the protective effect of DPx in BALB/_C_ mice that received thioacetamide (TAA) intraperitoneally for 6 weeks. Later DPx (20 mg/kg/day) or silymarin (50 mg/kg/day) was administered daily for 6 weeks, followed by biochemical and histological analyses of each group. Hematoxylin and eosin staining of the livers showed TAA-induced hepatic fibrosis, which was significantly reduced in the DPx group. DPx treatment significantly decreased TAA-induced hyperlipidemia as evidenced by the decreased AST, ALT, ALP, γ-GTP and serum TG concentrations and reduced the activities of catalase (CAT) and superoxide dismutase (SOD) activity. ELISA revealed reduced levels of total glutathione (GSH), malondialdehyde (MDA) and Inflammatory factors (IL-6, IL-1β, and TNF-α). Immunostaining showed reduced in collagen-1, α-SMA, and TGF-β1 expression and western blotting showed reduced levels of the apoptotic proteins, TGF-β1, p-Smad2/3, and Smad4. RT-qPCR and Western blotting revealed modifications in SIRT1, SIRT3 and SIRT4. Thus, DPx exerted a protective effect against TAA-induced hepatic fibrosis in the male BALB/_C_ mouse model by inhibiting oxidative stress, inflammation, and apoptosis via TGF-β1/Smads signaling.

## Introduction

Accumulation of extracellular matrix (ECM) proteins associated with alcoholic or non-alcoholic steatohepatitis may result in liver fibrosis 1-3. Hepatic fibrosis is generally reversible but may become irreversible, resulting in an end-stage process that leads to death 4. Morbidity and mortality rates attributed to hepatic fibrosis are continually increasing in developing countries 5, 6. In a global burden of disease report produced in 2013, cirrhosis ranked sixth in developed countries 5. Unfortunately, pharmaceutical companies have been unable to develop efficient anti-fibrotic medications, and the clinical management of liver fibrosis continues to be a challenge for researchers around the globe.

Hepatic fibrosis is associated with nodule formation and scar accumulation in the ECM 7, 8. Excess production of ECM proteins and accumulation of collagen-1 plays a crucial role in hepatic fibrogenesis 1, 3, 9. In addition, the ECM protein is directly correlated to hepatic stellate cell (HSC) activation and cytokine secretion from Kupffer cells, which are activated during liver fibrosis 10, 11. Myofibroblasts differentiate into fibroblasts, and TGF-β activation increases collagen synthesis during hepatic fibrosis 12-14. TGF-β1/Smad signaling pathway leads to the activation of HSCs by TGF- β1, resulting in the stimulation of fibroblasts 15, 16. TGF-β activates the c-Jun N-terminal kinase (JNK) pathway/stress-activated protein kinases (SAPKs)/p38 signaling cascade and non-Smad pathway. Upon stimulation, JNK/p38 and Smad, regulate apoptosis, whereas the activation of transcription factors leads to epithelial to mesenchymal transition (EMT) 17, 18.

Thioacetamide (TAA) induces hepatic fibrosis in rats and is used as a model in liver studies 19-22. Koblihová et al. found that bioactivation of TAA led to the formation of sulfene or sulfone metabolites via the CYP450 pathway. Metabolic activation of TAA produces reactive radicals in hepatocytes, causing oxidative stress and damaging necrotic tissues 22, 23. TAA-S-S-dioxide and TAA-sulfoxide are derivatives of TAA that produce lipid peroxides 24-26. TAA induces hepatic damage via lipid peroxidation and oxidative stress, which are processes responsible for the secretion of cytokines, prostaglandins, and profibrogenic growth factors 25, 26. In agreement with these findings, TAA-induced hepatic fibrosis is closely associated with oxidative stress in a rat model 19, 20. Therefore, TAA has been widely used to induce hepatic injury in Balb/C mice, resulting in severe liver fibrosis 27, 28. Although numerous strategies for managing hepatic fibrosis have been attempted 29, they generally have low efficacy and a high incidence of adverse effects, and new approaches for treating liver injury are urgently needed. Therefore, the development of alternative natural products has attracted interest, and plant-derived natural products have been sought for the treatment of hepatic fibrosis with minimal undesirable side effects. Extracts of traditional plants' stems, leaves, and roots are effective as anti-inflammatory, antioxidant, anti-complement, anti-cancer, and antidiabetic agents 30-35. *Dendropanax morbifera (D. morbifera)* extract has been used to treat menstrual, migraine, and muscle pain 36. The biologically active compounds derived from the stems, leaves, and roots of *D. morbifera* include polyphenols and polyacetylene compounds 32. In a previous study, we identified antidiabetic effects of *D. morbifera* extracts in rats 37. Dendropanoxide (DPx), which is an important component of *D. morbifera*, exhibited protective effects against cisplatin-induced acute kidney injury in rats. These data suggested that the mechanism underlying the protective action of DPx against nephrotoxicity involves antioxidant, anti-inflammatory, and apoptotic processes. However, the protective effects of DPx against hepatic fibrosis remain unclear. In this study, we used a TAA-induced mouse model of liver fibrosis to examine the possible effects of DPx. We compared the data generated using DPx with that produced using silymarin, is a clinically approved hepatoprotective drug in Asia and Europe 38.

## Materials and Methods

### Chemicals and materials

TAA and silymarin were obtained from Sigma Aldrich (St. Louis, Missouri, USA). The ELISA kits used to measure alanine aminotransferase (ALT), aspartate aminotransferase (AST), alkaline phosphatase (ALP) and r-glutamyl transferase (r-GPT) were acquired from Abcam (Cambridge, UK). Malondialdehyde (MDA), glutathione (GSH), catalase (CAT), and superoxide dismutase (SOD) assay kits were purchased from Cayman Chemical (Ann Arbor, Michigan, USA). Antibodies against collagen-1, TGF-β1, α-SMA, vimentin, Smad2/3, p-Smad2/3, Smad4, Smad7, p-PTEN, PTEN, p-Akt, Akt, p-PI3K, PI3K, and β-actin were purchased from Abcam (Cambridge, Massachusetts, USA) and Cell Signaling Technology (Danvers, Massachusetts, USA). Anti-mouse and anti-rabbit IgG and HRP-linked-conjugated secondary antibodies were procured from Santa Cruz Biotech. (Santa Cruz, California, USA).

### Extraction and isolation of DPx

*D. morbifera* was cultivated in Gwangyang, Jeollanam-do, Korea, and the aerial parts of the plant were collected in September 2018. Through the School of Pharmacy, Sungkyunkwan University, a voucher specimen of the plant (SKKU-Ph-18-012) was deposited. One hundred grams of the dried aerial part of *D. morbifera* was subjected to extraction twice with 1 L of water at 90^°^C for 5 h. The two extracts were mixed and concentrated under reduced pressure until 1Lof *D. morbifera* water extract was obtained. The aerial parts were cut and dried at 52^°^C for 12 h. Then 2.27 kg of the dried pieces were percolated twice with 95% ethanol (EtOH), once at room temperature for 24 h and once at 60°C for 5 h. The extracts were mixed, condensed, and heated to 40 degrees Celsius under decreased pressure. The ethanol extract (81.9 g) was dissolved in 1 liter water. Three organic solvents were used to fractionate the solution, yielding ethyl acetate (3.2 g), dichloromethane (21.1 g), n-butanol (7.2 g), and water (63.5 g) fractions. The dichloromethane (CH_2_Cl_2_) fraction was then collected and subjected to column chromatography. In total, 12 fractions were obtained using stepwise elution with CH_2_Cl_2_, hexane-CH_2_Cl_2_ (5:1, 3:1, and 1:1), and CH_2_Cl_2_-MeOH (100:1, 10:1, and 1:1). The 4^th^ fraction obtained using column chromatography was separated again using silica gel with hexane-CH_2_Cl_2_ solvent (5:1 and 1:1) a white amorphous dendropanoxide powder was obtained. Recrystallization was performed to purify DPx using a CH_2_Cl_2_-MeOH solvent mixture 37. The purity of DPx was measured using gas chromatography-mass spectrometry (GC-MS) and found to be 97.2%.

### Experimental design

**Figure 1:**
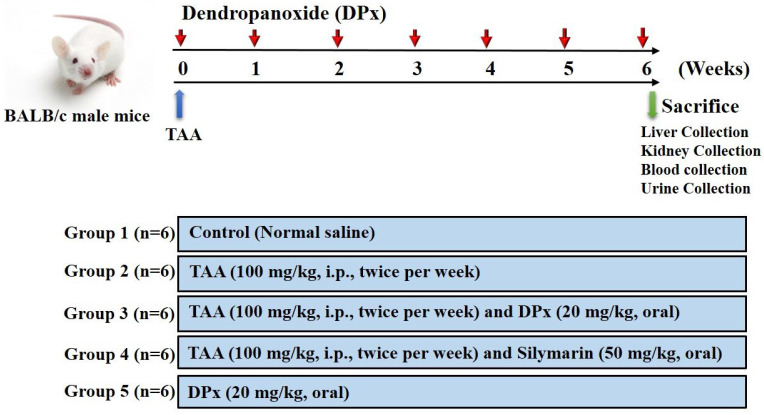
** Experimental design.** After 1 week of adaption, male Balb/C mice were divided randomly into 5 different groups: The control group was given normal saline water. Thioacetamide (TAA) was injected to induce liver fibrosis. Dendropanoxide (DPx) or Silymarin was administered in TAA-treated mice.

Male BALB/_C_ mice with a body weight of approximately 25± 5 g were obtained from Central Lab Animal Inc. (Seoul, Korea). All mice were housed in a room with a constant temperature (23 ± 0.5°C), relative humidity of 53-57%, and 12 h light/ dark cycle under specific pathogen-free conditions. All animals, they were housed in the laboratory for 10 days prior to the study. Hepatic fibrosis was induced in the animals by injecting TAA, as described in previous studies 19, 27, 39-41, with slight modifications (Figure 1). The animals were randomly divided into six groups 1) control; 2) TAA; 3) TAA + DPx; 4) TAA + Silymarin; and 5) DPx alone. To induce hepatic fibrosis, an intraperitoneal (i.p.) injection of TAA (100 mg/kg in normal saline) was administered to the group 2 mice twice per week for 6 weeks. Group 3 mice received i.p injection of TAA followed by DPx administration (20 mg/kg/day) via the oral route for 6 weeks. Group 4 received TAA via the i.p. route followed by silymarin (50 mg/kg/day) via oral route for 6 weeks. Only DPx (20 mg/kg/day) was administered to group 5 mice via oral gavage daily for 6 weeks. Laboratory animals (MIDF Publications No. 152785, revised 2018) involved in the experiments were handled and cared for according to the guideline of the Ministry of Food and Drug Safety Health. The Institutional Animal Care and Use Committee at Sungkyunkwan University approved the use of animals in this research (Approval Number SKKUIACUC-17-8-15). The mice were sacrificed 24 h after the last DPx treatment. The animals were given CO_2_ anesthesia on the last treatment day, and blood was drawn from the abdominal aorta using heparinized tubes. The blood was centrifuged at 3,000 x g for 15 min to separate the plasma. The livers of the animals were then removed. All samples were immediately stored at -80°C until they were used for histological and molecular studies.

#### Serum biochemical analysis

The serum samples were transferred to sterile tubes. Within 2 hours of collection, the samples were frozen at -80°C. The levels of AST, ALT, ALP, and γ-GTP were examined (Abaxis, Inc., Union City, CA). A UV-visible spectrophotometer (JASCO, V-650, Japan), was used to estimate the total cholesterol (TC), low-density lipoprotein (LDL), triglyceride (TG), high-density lipoprotein cholesterol (HDL-C), blood urea nitrogen (BUN) and creatinine levels at 505 nm. The samples fixed in paraffin were sliced into 3-5 µm sections, and overnight, 70% ethanol was used to dehydrate the sections. To detect any changes in the morphology of the liver tissue, Sirius red, Periodic acid-Schiff (PAS), and hematoxylin & eosin (H&E) were performed on the tissue sections. Masson's trichrome (MT) staining was performed to assess the histology and fibrosis. The microscopic images of the stained tissues were obtained using a light microscope (Zeiss Axiophot, Oberkochen, Germany) at 200× magnification.

### Determination of oxidative stress parameters

The GSH content was measured using commercially available glutathione detection kits (Enzo Biochem, Inc., NY, USA) as the manufacturer's guidelines. Liver tissue (approximately 100 mg) was homogenized in 5% metaphosphoric acid and centrifuged for 12 min at 12000 x g at 4°C. The supernatant from the homogenized tissue was collected and stored at 4°C. The reaction mixture was mixed with 10 µL of the diluted sample. The GSH levels are expressed as pmol/mg protein. The CAT enzyme assay measures the activity of the catalase enzyme by measuring its interaction with methanol in the presence of H_2_O_2_, which is a hazardous component of pathogenic ROS generation and regular aerobic metabolism (42). Formaldehyde production was measured using a colorimetric assay (Cayman Chemical Co., Ann Arbor, MI). The CAT content is as nmol/mg of protein. The amount of MDA was estimated using a colorimetric test kit (cat. no. KGE013) according to the manufacturer's guidelines. The SOD activity was calculated using colorimetric assay kits, (Cayman Chemical Co., Ann Arbor, MI), according to the manufacturer's guidelines. SOD activity was expressed as U/mg of protein.

### Western blotting analysis

Liver tissue (approx. 50 mg) was homogenized in 1 mL of RIPA lysis buffer. The homogenized mixture was centrifuged for 12 min at 12,500 × g at 4°C. The amount of protein in the supernatant was estimated using a bicinchoninic acid protein assay (Thermo Scientific, Pierce, MA, USA). The samples were vortexed and centrifuged at 10,000 × g for 20 min at 4 °C, and the supernatants were analyzed by western blot. Total proteins were resolved and denatured by boiling at 96°C with sample buffer for 5 min. The acquired samples were electrophoresed on SDS-PAGE gels (6-12%) and then electrotransferred onto an Immobilon-P PVDF membrane (Millipore, Burlington, MA, USA) in TBS-T (50 mM Tris-HCl (pH 7.6), 0.1% Tween-20 and 200 mM NaCl) solution containing 20% methanol. The PVDF membrane containing the transferred proteins was blocked in non-fat milk (5%) for 1 hour. The PVDF membranes were incubated with specific primary antibodies (1:1000) against collagen-1, TGF-β1, α-SMA, vimentin, Smad2/3, p-Smad2/3, Smad4, p-PTEN, PTEN, p-Akt, Akt, p-P38, P 38, SIRT1, SIRT3, SIRT4, and β-actin. The membranes were washed with TNT buffer for 30 min and treated with HRP-conjugated anti-rabbit IgG/anti-mouse IgG (1:20,000) secondary anti-bodies for an hour, washed with TNT buffer for 30 min, and dried for 60 min. Protein expression was visualized, and the band intensity was measured using a chemiluminescence (ECL)-plus kits (Amersham Biosciences Corp., Little Chalfont, UK).

### Analysis of TGF-β1

TGF-β1 levels were measured using an ELISA kit from R&D Systems (Minneapolis, Minnesota, USA) according to the manufacturer's instructions. Prior to the start of the experiment, equal volumes of 2.5 N acetic acid/10 M urea were added to the samples. The samples were diluted at a ratio of 1:24 to avoid matrix interference and transferred to 96-well plates coated with a specific TGF-β1 monoclonal antibody. The plates were incubated for an hour and washed. After washing, TGF-β1 conjugate (100 μL) was added to each well and incubated for 2 h at room temperature. The wells were washed, followed by addition of the substrate solution to the wells and incubating for 30 min (room temperature). Finally, the stop solution was added, and the optical density was measured at 450 nm.

### Analysis of proinflammatory cytokines

Proinflammatory cytokines such as IL-6, IL-1β, and TNF-α were detected at significantly higher concentrations in the serum of animals with hepatic fibrosis induced TAA. These proinflammatory cytokines were quantified using ELISA kits (Abcam, Cambridge, MA, USA) as per instructions provided by the manufacturer. The wells of a plate were coated with IL-6 specific antibodies, and the control, test and standard samples of IL-6 were added to these wells. The biotinylated antibody specific to IL-6 was incubated with the standards and samples at room temperature. HRP-conjugated streptavidin was then added, and wells were washed. After incubation, 3,3',5,5'-tetramethylbenzidine (TMB) solution was added. Finally, absorbance was measured at 450 nm using a spectrophotometer after adding the stop solution. The IL-1β and TNF-α levels were measured using the same method. The concentrations of these cytokines were determined using standard plot graphs.

### Hydroxyproline analysis

Hydroxyproline assay was used to measures the collagen levels in the tissue homogenates using an ELISA kit (Cell Biolabs, USA) according to the manufacturer's protocol. Liver tissue samples (100 mg) were homogenized in 5 N HCl (10 mL) and incubated overnight at 120°C. The following day, a 0.45 µm PVDF syringe filter (Sigma Aldrich) was used to filter the samples. The filtrate was vacuum evaporated for 45 min at 70°C. Multiple homogenates of the same sample were assayed, and the mean was calculated. The hydroxyproline levels were expressed as mg/g of protein.

### Immunohistochemical examination

The liver sections on the slides were deparaffinized and dehydrated using xylene and 100%, 95%, and 70% ethanol for 5 min. Antigen retrieval was performed by incubating the slides at 95-100°C in citrate buffer for 20 min. The tissue sections were treated with 3% H_2_O_2_ in methanol for 10 min to inactivate endogenous peroxidase activity. Primary antibodies such as α-SMA (1:200), TGF-β (1:300), and SIRT1 (1:1000), diluted with antibody dilution buffer, were left to interact with the tissue sections overnight at 4°C. After washing, the tissue sections were treated with biotinylated secondary HRP-streptavidin antibody for 30 min. at room temperature. DAB was used to visualize the labelled proteins using ultra-vision detection. Using a Zeiss microscope (Germany) coupled with a PAX-it!^TM^ software, the liver slices were observed and imaged at a magnification of 200X (PAXcam, Villa Park, USA).

### Immunofluorescence analysis

The liver sections were deparaffinized and dehydrated. Antigen retrieval was performed, and the tissues were permeabilized using ice-cold 100% methanol for 10 min, followed by blocking of the tissues with 10% normal goat serum for one hour. The slides were subsequently treated with rabbit polyclonal antibodies for TGF-β1, collagen-1, and α-SMA overnight at room temperature (diluted in PBS at 1:100). After washing with PBS, the liver sections, were incubated with fluorochrome-conjugated secondary antibody (anti-rabbit IgG-TRITC) in the dark. The nuclei were counter-stained using DAPI for 2-3 min and washed. The slides were imaged using an Olympus FV500 laser confocal microscope (Olympus, Tokyo, Japan). The red area stained with TRITC was positively stained and measured using FluoView Olympus version 4.0 (Tokyo, Japan).

### TUNEL assay

TUNEL assays were performed to detect DNA fragmentation during apoptosis in paraffin-embedded tissue sections. TUNEL assays involve fixation and permeabilization of the tissue, followed by in addition of TUNEL reagents. Apoptotic cells were analyzed using the DeadEndTM colorimetric system (Promega, Madison, WI, USA).

### RT-PCR analyses

Total RNA from the liver tissues was extracted using TRIzol reagent (15596018, Life Technologies, CA, USA). The extracted RNA was quantified using a NanoDrop analyzer. Maxime RT PreMix (catalogue no. 25081/96, Intron Biotech, Seoul, South Korea) with an oligo-dT primer was used to synthesize cDNA. PCR amplification was performed using the synthesized cDNA as a template. SYBR Premix Ex Taq II kits were used to perform quantitative RT-PCR with 20 μL of the mixture. The cycling conditions were as follows: initial denaturation at 95°C for 3 min, followed by 35 cycles of 95°C for 30 s, 55°C for 30 s, and 72°C for 30 s, and a final extension at 72°C for 5 min. β-Actin was used as the reference control gene.

### Statistical analysis

Data from the experiments are presented as mean SD ± (n = 6). One-way analysis of variance (ANOVA) was used to compare mean values, and Turkey's honest significant difference (HSD) post-hoc test was used for multiple comparisons. Graph Pad Prism v5.0 software (GraphPad Software, San Diego, California USA, www.graphpad.com) was used for statistical analysis. The data were considered statistically significant when the p-value was <0.05.

## Results

### Effects of DPx on TAA-induced body and liver weight changes in BALB/_C_ mice

**Figure 2:**
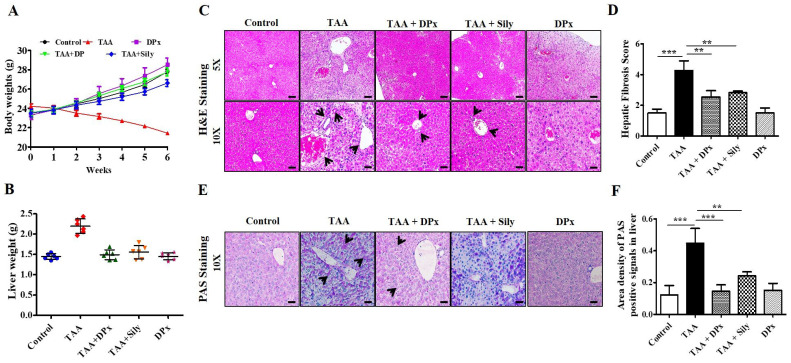
** Protective effects of DPx on TAA-induced hepatic fibrosis**. TAA (100 mg/kg) was injected into male Balb/C mice twice a week for 6 weeks with or without DPx/Silymarin administration. The mice received DPx (20 mg/kg/day) or silymarin (50 mg/kg/day) for 6 weeks by oral gavage. DPx (20 mg/kg/day) and Silymarin (50 mg/kg/day) were administered to the mice daily by oral gavage for 6 weeks. Effects of DPx on changes in liver morphology. (A) Effect of DPx or Silymarin on changes in body weight and (B) liver weights. (C) Hematoxyline & eosine (H&E)-stained sections of the liver displaying the histology of mice from the experimental group. Histological alterations indicate an increase in fibrous tissue thickness, degenerative hepatocytes, enlarged portal tract, hepatic central vein and hepatic nodule increases (cirrhosis). CV, central vein; PV, portal vein. (D) Histological score of H&E staining of liver section. (E) Effect of DPx or Silymarin on glycogen content in the hepatocyte analyzed using periodic acid-schiff (PAS) staining. (F) PAS staining score of liver sections. Images depicting stained section from three animals from each experimental group. (Magnification 200X; bar=100 μm). Each value represents the mean standard deviation of 6 mice per group. Statistical analysis was performed by one-way ANOVA followed by Tukey's (honest significant difference (HSD) post hoc test for multiple comparisons. (***p < 0.001, **p < 0.01, *p < 0.05).

Food intake (g/body weight) capacity was not altered among the groups (data not shown). Treatment with TAA caused severe morphological alterations in the livers of BALB/_C_ mice, whereas DPx treatment mitigated the morphological alterations. Body weight gain was significantly lower in the TAA-treated group than untreated control group. No changes in body weight were observed after treatment with DPx or silymarin (Figure 2A). Similarly, the relative liver weight was significantly increased in the TAA-treated group, whereas DPx or silymarin protected against TAA-associated alterations in liver weight (Figure 2B).

### Histological effect of DPx on the liver of TAA-treated BALB/_C_ mice

Histopathological outcomes of the liver showed typical architecture associated with portal tracts and the central vein of the liver lobule in control mice. However, TAA-treated mice displayed severely increased necrosis around the connective tissue and central vein. Another critical alteration in the liver tissue of TAA-treated mice was observed in the degenerative and inflammatory infiltrates. However, the morphological abnormalities of the liver in TAA-treated mice were dramatically protected by the administration of DPx or Silymarin (Figure 2C and 2D). Furthermore, PAS staining also showed improved glycogen storage in the livers of mice treated with DPx or silymarin (Figure 2E and 2F).

### Effects of DPx on serum biochemical parameters in TAA-treated BALB/_C_ mice

**Figure 3:**
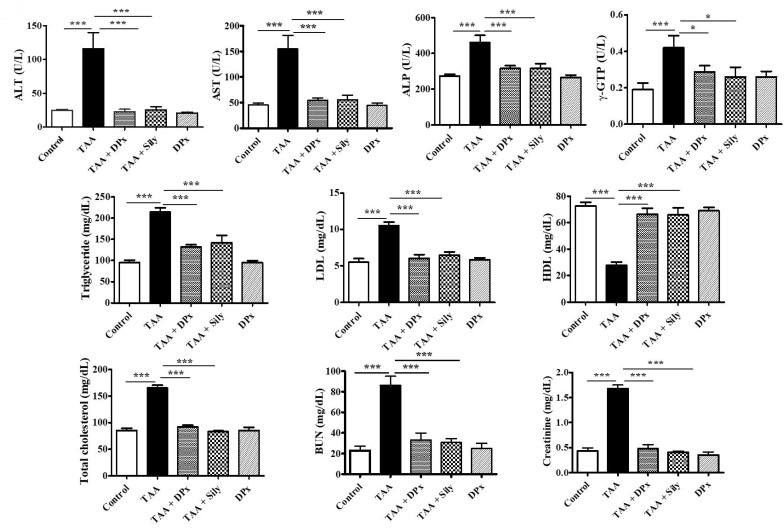
** Effects of DPx on thioacetamide (TAA) induced hepatic fibrosis**. TAA (100 mg/kg) was injected into male BALB/_C_ mice twice a week for 6 weeks with or without DPx or Silymarin administration. The mice received daily oral gavage doses of DPx (20 mg/kg/day) or Silymarin (50 mg/kg/day) for 6 weeks. Effect of DPx or Silymarin on serum aspartate aminotransferase (AST), alanine aminotransferase (ALT), alkaline phosphatase (ALP), r-glutamyl transferase (r-GPT), triglyceride (TG), high-density lipoprotein (HDL), low-density lipoprotein (LDL), total cholesterol (TC), blood urea nitrogen (BUN) and creatinine activities. The values are expressed as mean ± S.D. of six mice per group. Statistical analysis was performed by one-way ANOVA followed by Tukey's (honest significant difference (HSD) post hoc test for multiple comparisons. (***p < 0.001, **p < 0.01, *p < 0.05).

Serum enzymatic biomarker analysis revealed that the TAA-treated group had significantly higher AST, ALP, ALT, and r-GPT levels. However, levels of these enzymatic biomarkers were considerably reduced after treatment with DPx or Silymarin (Figure 3). The TAA-treated group showed higher levels of critical regulators associated with hepatic damage such as LDL, TC, and TG than did the control group. Conversely, the TAA-treated group showed an apparent increase in BUN and creatinine levels compared with that of the control group. However, the serum biochemical parameters in the TAA-treated groups were markedly decreased in mice treated with DPx or Silymarin. The alteration of HDL was significantly restored, following treatment with DPx or Silymarin (Figure 3).

### Effect of DPx on oxidative stress markers in TAA-treated BALB/_C_ mice

**Figure 4:**
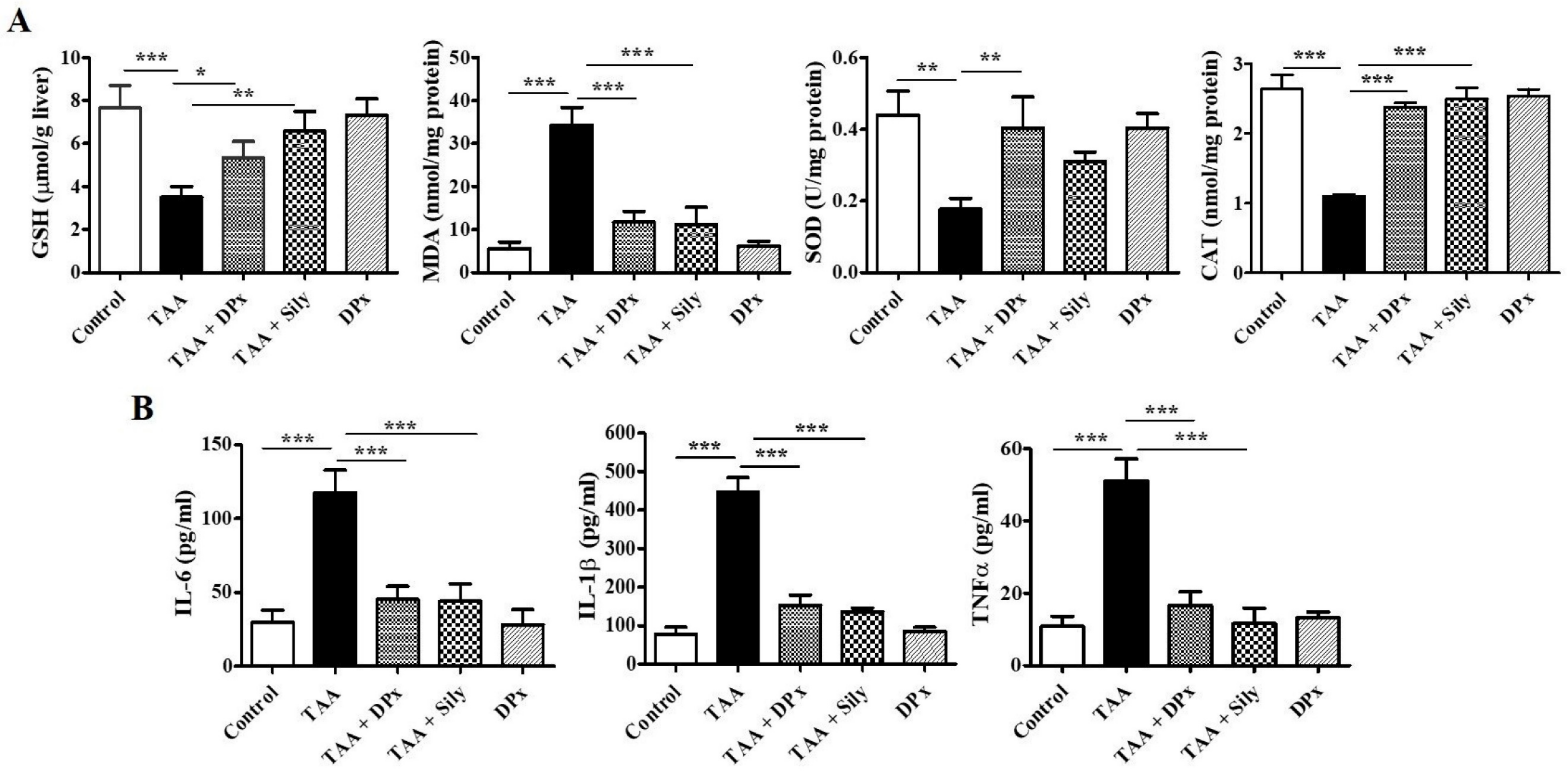
** Effects of DPx on the activity of antioxidant enzymes in the liver. Thioacetamide (TAA) was injected (100 mg/kg) into male BALB/_C_ mice twice a week for 6 weeks with or without DPx and Silymarin administration.** The mice received daily oral gavage doses of DPx (20 mg/kg/day) or Silymarin (50 mg/kg/day) for 6 weeks. (A) The GSH, SOD, catalase and MDA concentrations were analyzed in the liver tissue. SOD, Superoxide dismutase; GSH, Glutathione; MDA, Malondialdehyde; CAT, catalase. (B) Effect of DPx on the serum of proinflammatory cytokines in the BALB/_C_ mice with TAA-induced liver fibrosis., The levels of proinflammatory cytokines (IL-6, IL-1β and TNF-α) significantly decreased in mice with liver fibrosis when treated with DPx. The values are expressed as mean ± S.D. of six mice per group. Statistical analysis was performed using one-way ANOVA followed by Tukey's (honest significant difference (HSD) post hoc test for multiple comparisons. (***p < 0.001, **p < 0.01, *p < 0.05).

To evaluate the antioxidant status, the GSH, SOD, MDA, and CAT levels were measured in mice with TAA-induced liver fibrosis. Mice treated with TAA had considerably lower GSH levels in the liver; however, the level increased, following treatment with DPx or Silymarin. Furthermore, SOD and CAT enzyme activities were sharply reduced in TAA-induced liver fibrosis, whereas treatment with DPx restored SOD and CAT activities in the liver of TAA-treated mice. Hepatic fibrosis was confirmed using allied with oxidative stress markers such as MDA, which are responsible for lipid peroxidation in the liver. These data revealed that MDA content was significantly increased in mice with TAA-induced liver fibrosis mice. In contrast, a significant reduction in MDA levels was observed following treatment with DPx or silymarin (Figure 4A).

### Effect of DPx on inflammatory cytokines mediators in TAA-treated BALB/_C_ mice

Excessive fat accumulation in the liver can lead to inflammation. The concentrations of proinflammatory cytokines including IL-6, IL-1β, and TNF-α were assessed in the serum. As shown in Figure 4B, IL-6, IL-1β, and TNF-α level were significantly increased in TAA-treated mice compared with that in the control group, whereas DPx or Silymarin treatment reduced the levels of the cytokines in TAA-treated mice. These data indicate that DPx inhibits the expression of proinflammatory cytokines.

### Effects of DPx on cell death or apoptosis in the liver of TAA-treated BALB/_C_ mice

**Figure 5:**
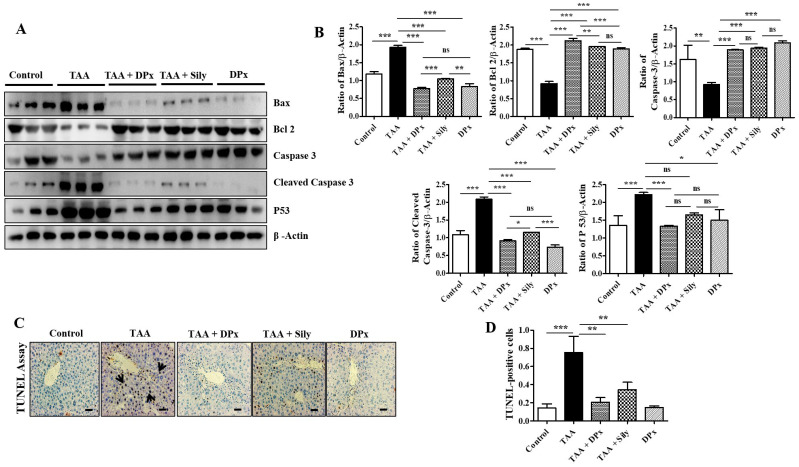
** Effects of DPx on hepatic fibrosis induced by the intraperitoneal (i.p) injection of thioacetamide (TAA) (100 mg/kg) in male Balb/C mice twice a week for 6 weeks with or without DPx or Silymarin administration.** The mice received daily oral gavage doses of DPx (20 mg/kg/day) or silymarin (50 mg/kg/day) for 6 weeks. (A) Western blotting was performed to analyze of BAX, Bcl-2, caspase 3, cleaved caspase-3, and p53 protein levels in the liver tissues. (B) The image J program used densitometry to analyze band intensity. (C) The TUNEL assay was used to assess the level of apoptosis in mice's livers following TAA administration. Arrowheads indicate the markers for TUNEL-positive cells. (D) Score index of TUNEL-positive cells. The values are expressed as mean ± S.D. of six mice per group. Statistical analysis was performed using one-way ANOVA followed by Tukey's (honest significant difference (HSD) post hoc test for multiple comparisons. (***p < 0.001, **p < 0.01, *p < 0.05).

Hepatic stellate cells migrate to the site of injury following exposure to TAA, where they engulf apoptotic bodies in the hepatocytes that have undergone apoptosis 43. In the liver of TAA-treated mice, the expression of Bcl-2 was markedly decreased, whereas that of Bax, p53, and cleaved caspase-3 was increased. However, the fibrotic liver was significantly protected as evidenced by the restoration of the expression of the proteins responsible for apoptosis (Figure 5A and 5B). These results indicate that DPx or Silymarin can potentially control TAA-induced liver damage. In connection with the above mechanistic approach, we performed TUNEL assay to quantify chromatin condensation, which is related to the severity of liver damage. Massive hepatocyte apoptosis was noticed in TAA induced mice livers. However, the apoptosis was markedly counter acted by administration with DPx or Silymarin (Figure 5C and 5D).

### Effect of DPx on ECM production and accumulation in the liver of TAA-treated BALB/_C_ mice

**Figure 6:**
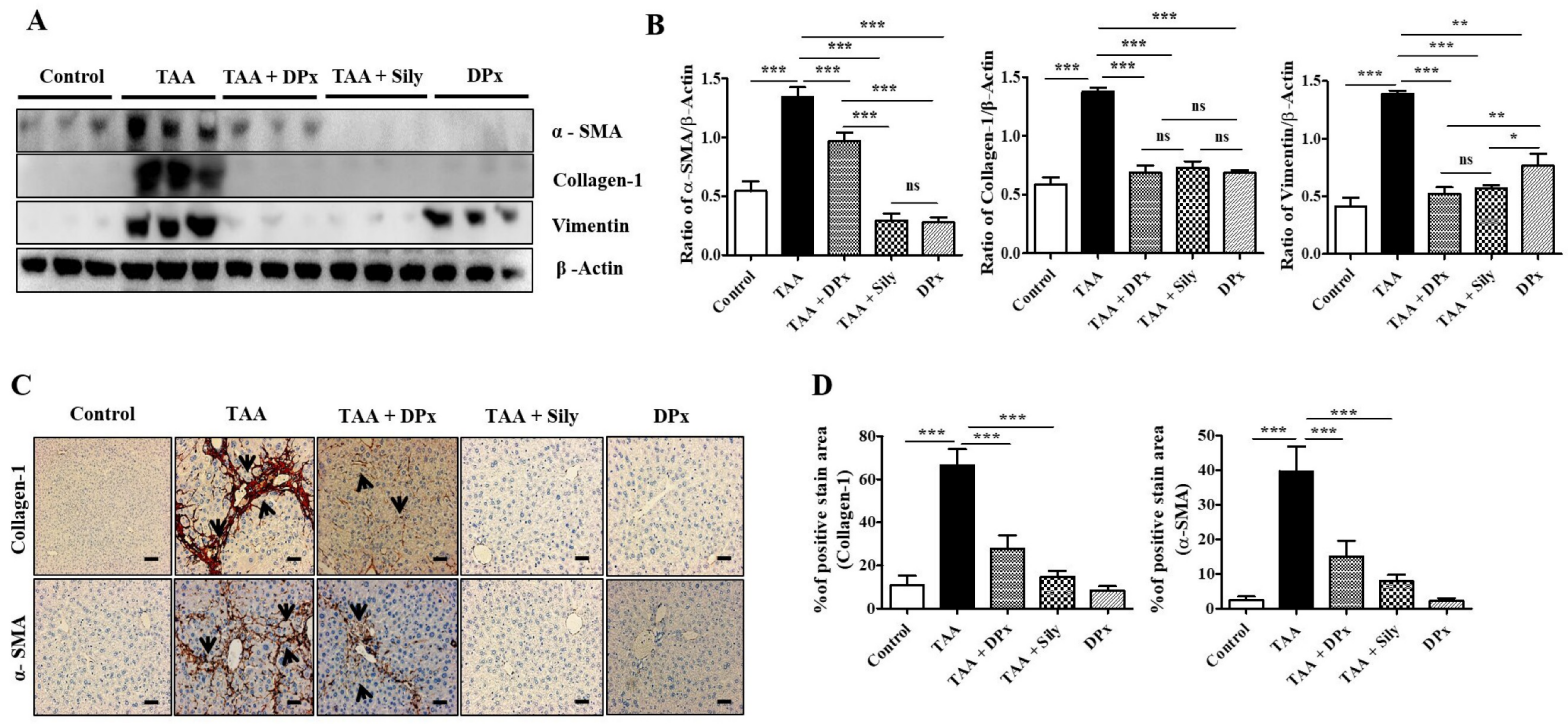

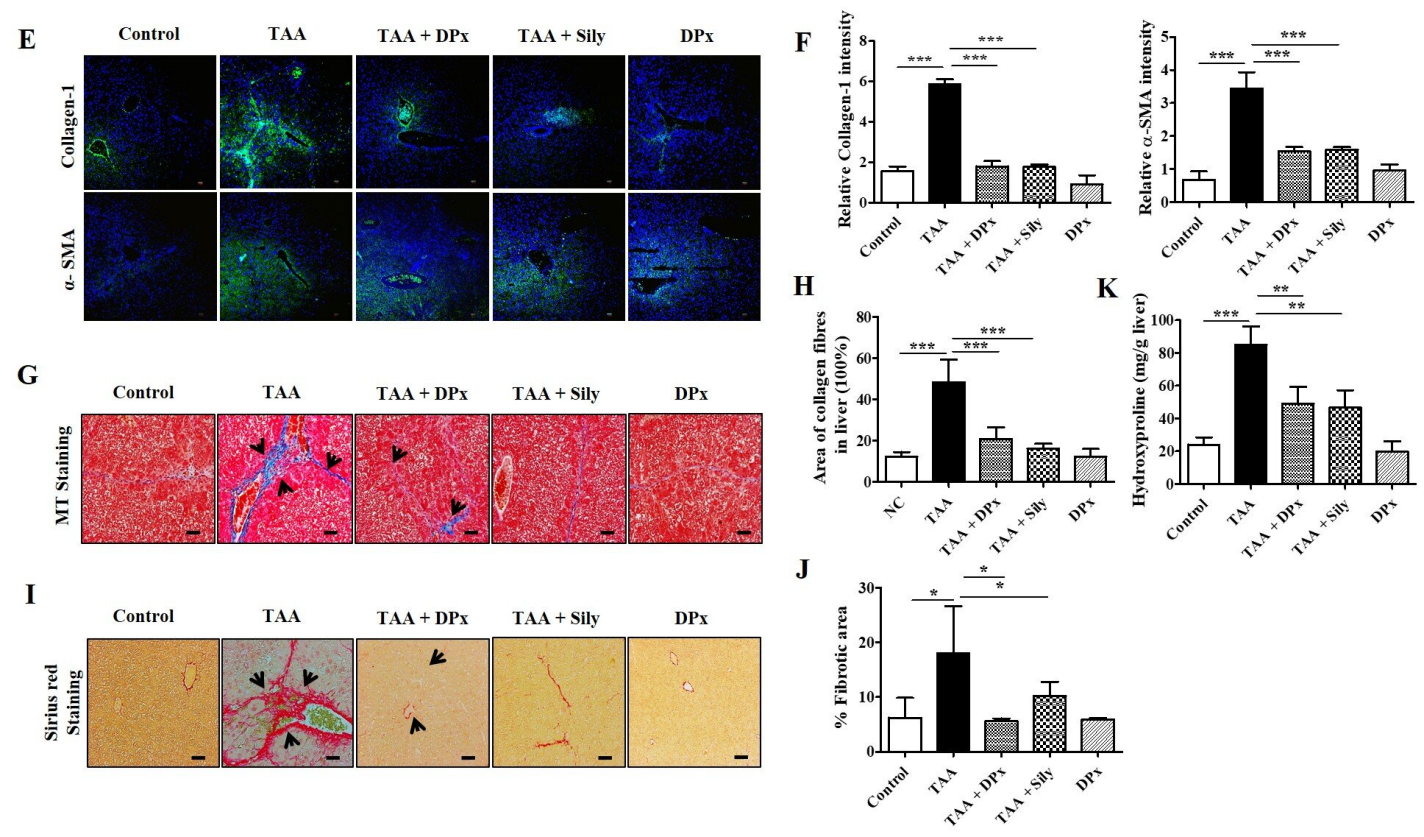
** Role of DPx on extracellular matrix (ECM) proteins, hydroxyproline content and histopathological changes in thioacetamide (TAA)-induced liver fibrosis.** TAA (100 mg/kg) was injected into male BALB/_C_ mice twice a week for 6 weeks with or without DPx or Silymarin administration. The mice received daily doses of DPx (20 mg/kg/day) or silymarin (50 mg/kg/day) via oral gavages for a period of 6 weeks. (A) The expression level of the extracellular matrix proteins such as α-SMA, collagen-1 and vimentin were measured using western blotting analysis in the TAA induced liver fibrosis model. β-Actin was used as the loading control. (B) Image J densitometry was used to analyze the band intensity. (C) Representative immunohistochemical staining of α-SMA and collagen-1. (D) Percentage of areas (%) that stained positively for collagen-1 and α-SMA. (E) Immunofluorescence staining of collagen-1 and α-SMA in the liver of the TAA-treated mice. (F) The relative intensity of collagen-1 and α-SMA was analyzed densitometrically using the Image J software. (G) Masson's trichrome (MT) staining. (H) Percentage of area (%) with stained collagen fibers in the liver. (I) Sirius red staining. (J) Percentage of area (%) representing collagen in the liver. (K) The concentration of 4-hydroxyproline in the liver of experimental groups. The values are expressed as mean ± S.D. of six mice per group. Statistical analysis was performed by one-way ANOVA followed using Tukey's (honest significant difference (HSD) post hoc test for multiple comparisons. (***p < 0.001, **p < 0.01, *p < 0.05).

Vimentin, collagen-1, and α-SMA protein expression levels were markedly increased in the livers of TAA-treated animals, which were significantly lowered on treatment with DPx and silymarin (Figure 6A and B). Immunohistochemical staining showed that exposure to DPx or silymarin after TAA treatment reduced the number of collagen-1 and α-SMA positive cells in the sinusoids and fibrous septa in the livers of the mice (Figure 6C and 6D). These results suggest that TAA-associated ECM accumulation was significantly attenuated by DP and silymarin treatment. Similarly, the immunofluorescence staining of collagen1 and α-SMA in TAA-induced fibrotic liver was consistent with the result of immunoblot analysis and IHC staining (Figure 6E and 6F). To confirm the protective effect of DPx against on TAA-induced hepatic fibrosis, Masson's trichrome (MT) staining was performed to detect collagen accumulation. A significantly higher level of collagen associated with nodular formations was observed in the hepatocytes of TAA-treated mice. Additionally, the collagen surrounding the extracellular space, particularly in the portal triad, increased dramatically in the TAA-treated mice. Surprisingly the blue-stained collagen content was increased in the lobules, and extracellular part, mainly in the portal triad of TAA-treated mice. This effect was significantly attenuated in the TAA-treated mice, followed by DPx or silymarin treatment (Figure 6G and 6H). Massive collagen accumulation in Sirius Red-stained livers was noticed in the TAA-treated mice compared with that in the control mice. However, DPx treatment significantly reduced collagen deposition (Figure 6I and 6J). One distinctive amino acid used in collagen formation is hydroxyproline, which indicates the degree of liver fibrosis 44. A higher level of hydroxyproline was detected in mice with TAA-induced liver fibrosis than in contrast with normal control mice. Moreover, the administration of DPx or silymarin significantly reduced the hydroxyproline content in the livers of TAA-treated mice (Figure 6K).

### Effect of DPx on the expression of SIRTs in the liver of TAA-treated BALB/_C_ mice

**Figure 7:**
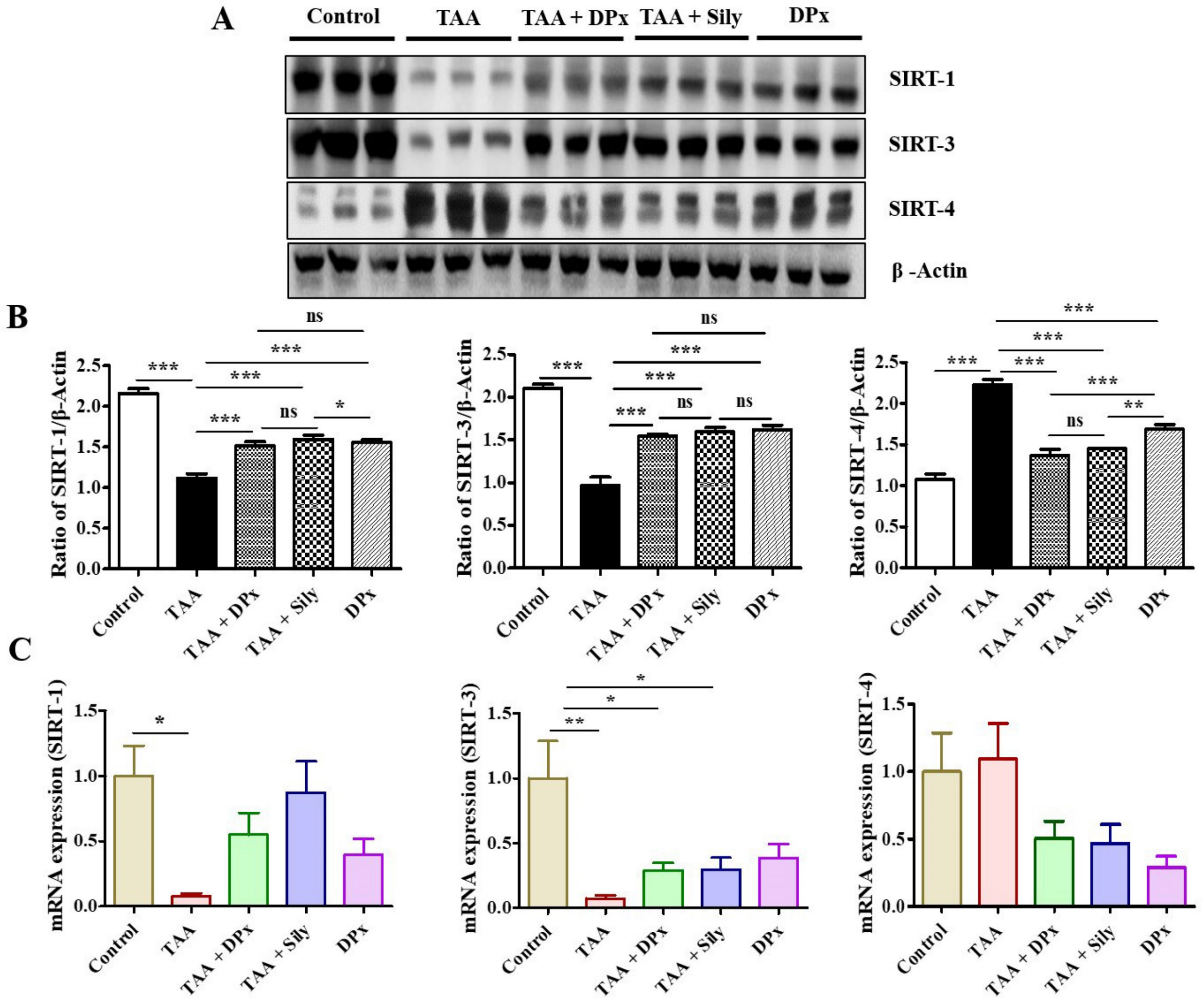
** The effect of DPx on SIRT expression in the hepatocytes of mice with thioacetamide (TAA)-induced liver fibrosis mice was examined using western blotting analysis and the mRNA expression was determined using polymerase chain reaction (PCR).** TAA (100 mg/kg) was injected into male Balb/C mice twice a week for 6 weeks with or without DPx or silymarin administration. The mice received daily oral gavage doses of DPx (20 mg/kg/day), and silymarin (50 mg/kg/day) for 6 weeks. (A) Representative image of the western blotting analysis of SIRT1, SIRT3 and SIRT4 in TAA-induced experimental mice model. (B) Image J densitometry was used to analyze the band intensity. (C) The mRNA expression levels of the SIRTs (SIRT1, SIRT3, and SIRT4) were examined using qPCR. The values are expressed as mean ± S.D. of six mice per group. Statistical analysis was performed by one-way ANOVA followed by Tukey's (honest significant difference (HSD) post hoc test for multiple comparisons. (***p < 0.001, **p < 0.01, *p < 0.05).

The experimental data showed that the expression of SIRT1 and SIRT3 in TAA-treated mice was much lower than that in the normal control mice. Additionally, the expressions of SIRT1 and SIRT3 increased in TAA-induced fibrosis liver following DPx or silymarin treatment. In contrast, SIRT4 level in the TAA-treated group was higher the TAA-treated group than that in the normal control group. However, treatment with DPx or silymarin attenuated SIRT4 expression in the liver of TAA-treated mice (Figure 7A and 7B). qPCR analysis was performed to corroborate the SIRT expressions data with previously obtained data. Supporting the western blot results, the mRNA expression of SIRT1 and SIRT3 was also decreased in the TAA-treated mice. The mRNA levels were restored after the treatment with DPx or silymarin in the TAA-treated mice. Similarly, SIRT4 expression was increased in the group treated with TAA and was attenuated by the treatment with DPx or silymarin (Figure 7C).

### DPx attenuated liver injury via TGF-β/Smads signaling pathway in the TAA-treated BALB/_C_ mice

**Figure 8:**
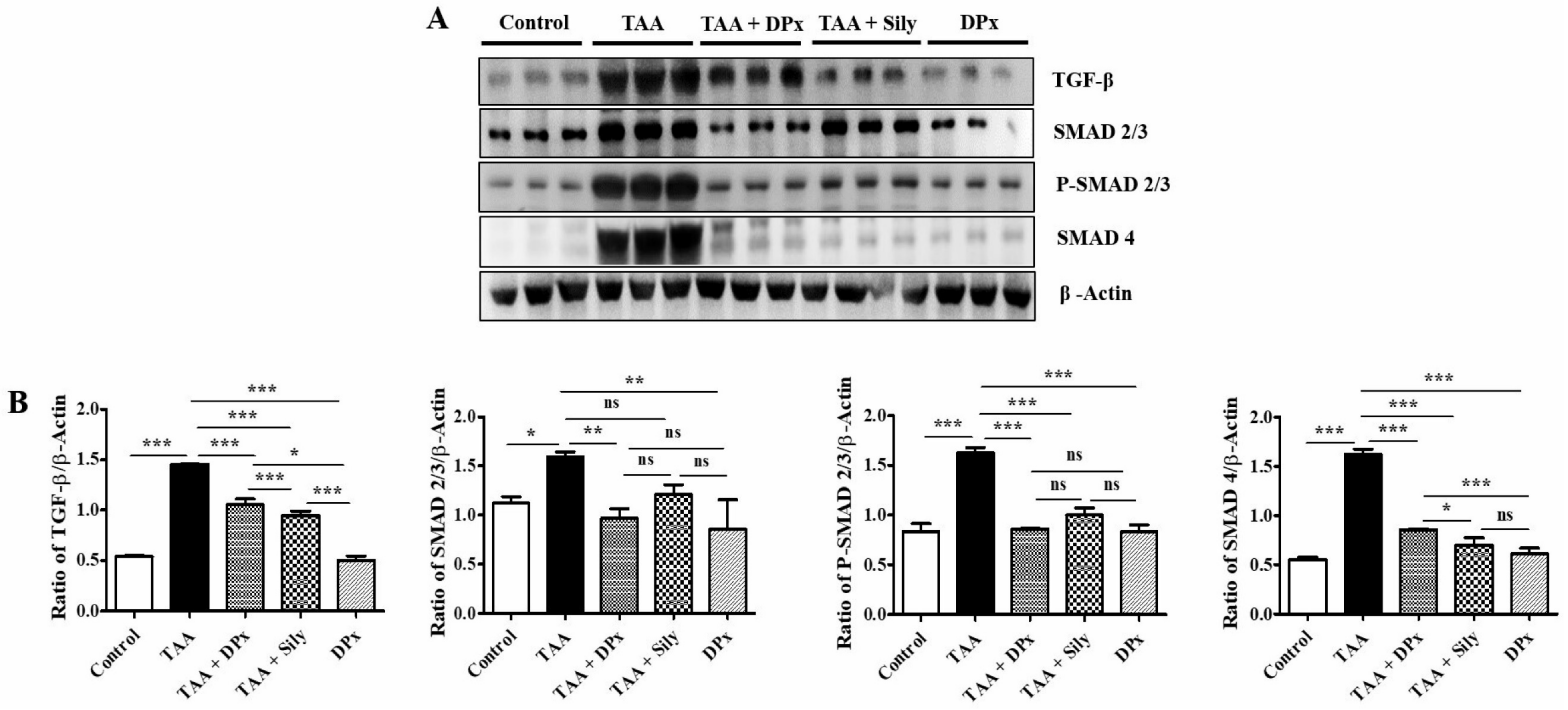

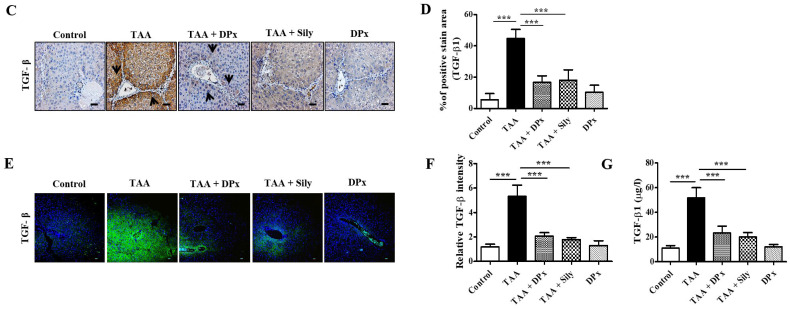
** Effects of DPx on the expression of TGF-β1, Smad2/3, and Smad4 in the livers of Balb/C mice treated with thioacetamide (TAA).** TAA (100 mg/kg) was injected into male Balb/C mice twice a week for 6 weeks with or without DPx or Silymarin administration The Mice received daily oral gavage doses of DPx (20 mg/kg/day), and silymarin (50 mg/kg/day) for a period of 6 weeks. (A) Western blotting analysis of TGF-β1, Smad2/3, p-Smad2/3, and Smad4 in liver tissue. (B) Image J densitometry was used to analyze the band intensity. (C) Representation of the immunohistochemical staining of TGF-β1. (D) Percentage (%) of positively stained area with respect to TGF-β1. (E) Representation of the immunofluorescence staining of TGF-β1 in the liver of the TAA-induced experimental mice model. (F) The relative intensity of TGF-β1 was analyzed densitometrically using ImageJ software. (G) ELISA results showing the concentration of TGF-β1 in the serum of experimental groups. The values are expressed as mean ± S.D. of six mice per group. Statistical analysis was performed by one-way ANOVA followed using Tukey's (honest significant difference (HSD) post hoc test for multiple comparisons. (***p < 0.001, **p < 0.01, *p < 0.05).

To determine the ameliorative effect of DPx in the treatment of liver fibrosis, TGF-β1 expression in the mouse serum was examined. TGF-β1 expression in the liver was analyzed using immunoblotting. Smad2/3 is an important regulator of the TGF-β1 signaling pathway. The phosphorylation of Smad2/3 plays an important role in blocking the downstream signaling pathway during the development of liver fibrosis 45. To investigate the function of DPx in the TGF-β1/Smad2/3 pathway, we measured Smad2/3 expression in the liver using western blotting analysis. The data showed that p-Smad2/3 expression was upregulated in the TAA-treated group. DPx and silymarin treatment significantly reduced the phosphorylation of Smad2/3 in the TAA-treated mice. In contrast, the expression of Smad4 associated with the TGF- β1/Smad2/3 signaling pathway was decreased in the TAA-treated group, following DPx administration (Figure 8A and 8B). Immunohistochemical analysis was performed to detect TGF-β1 expression in the liver. In the TAA-treated group, TGF-β1 expression was significantly increased in the liver section. However, TGF-β1 expression was significantly declined by DPx or silymarin treatment (Figure 8C and 8D). Similar results were observed in the immunofluorescence analysis (Figure 8E and 8F). In addition, the concentration of TGF-β1 in the serum was analyzed using ELISA. TGF-β1 levels in the serum were noticeably higher in the TAA-treated group than in the control group, whereas DPx and silymarin treatment dramatically reduced TGF-β1 levels in the serum of TAA-treated animals (Figure 8G).

### DPx restored the expression of p-Akt and p-PTEN in the livers of TAA-treated BALB/_C_ mice

**Figure 9:**
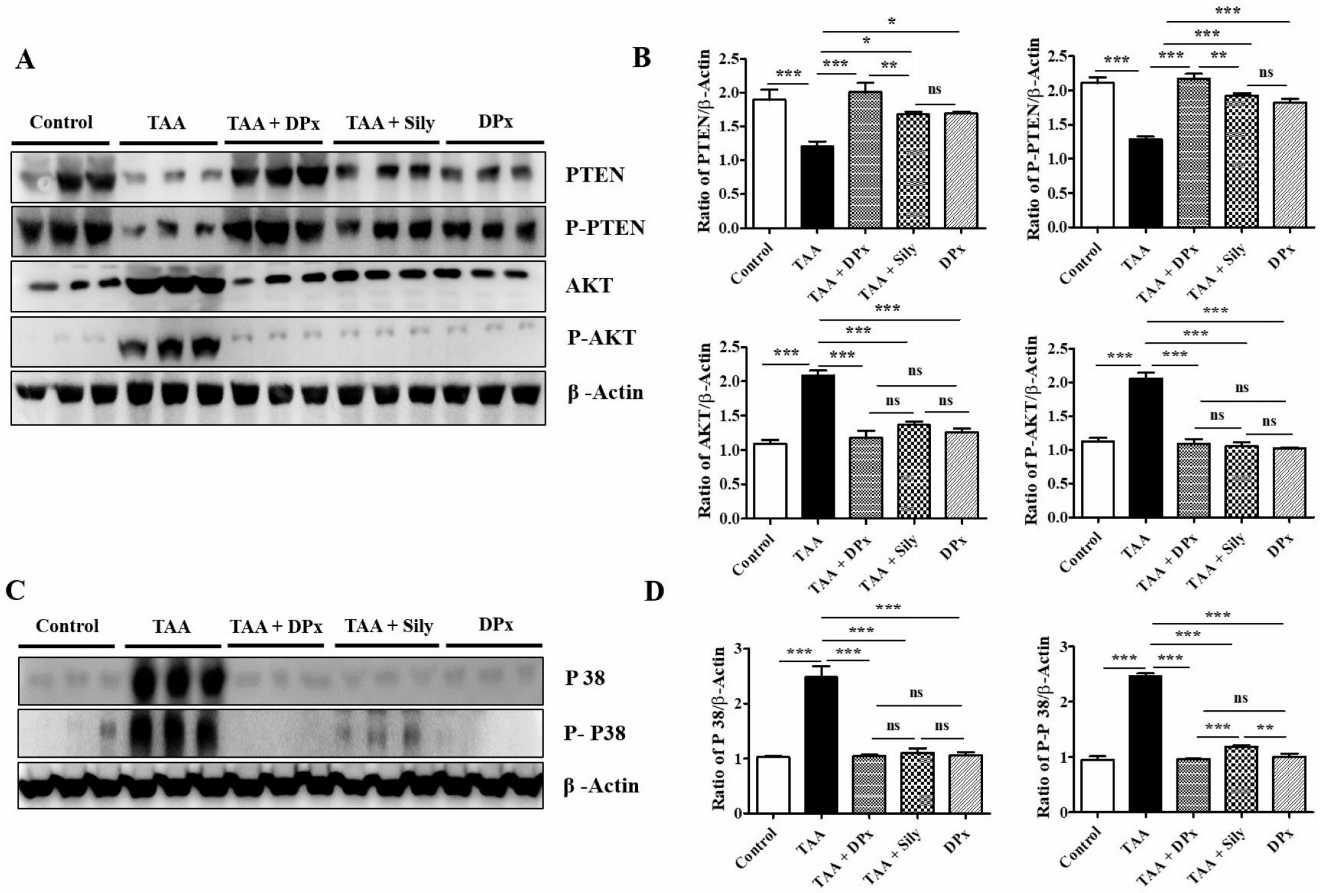
** Effects of DPx on the expression of p-PTEN, p-Akt and p38 in the liver of thioacetamide (TAA)-treated BALB/_C._ mice**. TAA (100 mg/kg) was injected into male Balb/C mice twice a week for 6 weeks with or without DPx or Silymarin administration. The mice received daily oral gavage doses of DPx (20 mg/kg/day), and silymarin (50 mg/kg/day) for six weeks. (A) PTEN, p-PTEN, protein kinase B (Akt), and p-Akt expression levels were determined using western blotting analysis in an experimental model of TAA-induced hepatic fibrosis. (B) Image J densitometry was used to analyze the band intensity of the western blots (C) Western blot bands of p38 and phosphorylated p38 from the liver tissues of TAA-treated mice were analyzed. (D) Image J densitometry was used to analyze the band intensity of p38 and phosphorylated p38. The values are expressed as mean ± S.D. of six mice per group. Statistical analysis was performed using one-way ANOVA followed by Tukey's (honest significant difference (HSD) post hoc test for multiple comparisons. (***p < 0.001, **p < 0.01, *p < 0.05).

We investigated the role of the Akt and TGF-β/SMAD pathways in the progression of liver fibrosis. p-Akt expression was significantly upregulated in TAA-treated mice compared with that in control mice. However, p-Akt expression was downregulated, following by the administration of DPx or silymarin. The expression of p-PTEN which is a regulator of p-Akt, was reserved. TAA-treated mice exhibited a significant reduction in p-PTEN protein levels, which were increased by DPx or silymarin treatment (Figure 9A and 9B). The expression of p38 protein involved in the TGF-β/non-SMAD3 signaling pathway was downregulated in TAA-treated mice, after DPx administration (Figure 9C and 9D).

## Discussion

The pathophysiology of liver fibrosis is associated with oxidative stress and inflammatory processes. These two critical phenomena occur following TAA administration. These data demonstrated that TAA treatment resulted in liver enlargement in mice. Similarly, daily TAA administration for 8-10 weeks significantly elevated the hepatic weight in male C57BL/6 mice 39. Interestingly TAA also increased liver weight in male Sprague-Dawley rats 20. Typical signs of TAA-induced hepatic fibrosis included a decrease in body weight and increased relative weight of the liver 22, 23. Friedman (2008) indicated that a higher level of production and accumulation of ECM proteins in hepatocytes might result in higher liver weight following TAA treatment 11. Interestingly, treatment with DPx significantly reduced the TAA-mediated increase in liver weight, in agreement with the results of a previous study 20.

In this study, silymarin was used as a positive control to assess its effect on TAA-treated mice. This study demonstrated the protective effects of dendropanoxide against TAA induced toxicity. However, the effects of DPx were only slightly different from that of silymarin, which is a common hepatoprotective drug, with superior mitigating qualities as that of DPx. The flavonoid combination in silymarin possesses hepatoprotective properties and functions as an antioxidant in rats with oxidative damage caused by cadmium exposure 46, 47. Oral administration of silymarin considerably enhanced the antioxidant defense in the liver compared with that in the liver of animals treated with cadmium 48. Additionally, the effect of silymarin (50 mg/kg) on TAA induced liver fibrosis induced by TAA in Sprague-Dawley rats was assessed 49. Silymarin exerts its effects through a variety of underlying mechanisms making it a hepatoprotective agent. Silymarin scavenges free radicals and increases glutathione levels, inhibiting lipid peroxidation 50, 51. Silymarin's steroid-like effect by acting on RNA polymerase can encourage the regeneration abilities of liver cells. As a results ribosomal RNA production increases and DNA synthesis increases. This promotes protein production and helps heal damaged cells 52. Silymarin also controls and stabilizes the permeability of the hepatocyte membrane, preventing xenobiotics from penetrating hepatocytes 53. The anti-inflammatory effects of silymarin also reduce inflammatory cytokine production and consequent inflammation, which reduces hepatic tissue damage 54, 55. Thus, silymarin is a powerful drug that to protects the liver against toxins owing to its variety of qualities.

Serum ALT, AST, ALP, and γ-GTP levels are distinct features of liver fibrosis. Previous studies have indicated that serum enzyme activities are significantly increased, following exposure to TAA 56, 57. In agreement with other studies, our results revealed that ALT, AST, ALP, and γ-GTP activities were increased significantly in mice with TAA-induced liver fibrosis, whereas treatment with DPx reversed all abnormalities indicated by hepatic damage biomarkers. Insulin resistance, hepatic steatosis, and fibrosis impair glycogen synthesis 58, 59 and improved glucose tolerance 60 results from enhanced liver glycogen synthesis without regard to insulin signaling 61, 62. The failure to manufacture liver glycogen results in increased lipid synthesis, ectopic liver fat deposition, and liver-specific insulin signaling dysfunction. According to a recent study, mice with little liver glycogen developed steatosis and hepatic insulin resistance 63.

The risk of liver fibrosis increase with excessive hepatic glycogen storage, liver injury, inflammation, and collagen deposition 64. The amount of glucose stored as glycogen determined using PAS staining, which is used to assess glycogen storage function. In our study, the TAA group showed a higher PAS-positive area (magenta), indicating the presence of abundant glycogen. However, mice treated with DPx or Silymarin showed less PAS-positive tissues. This suggests that the administration of DPx or Silymarin reduced the amount of glycogen stored in TAA-treated mice. Accumulation of TG in hepatocytes is the primary criterion for the development of liver fibrosis. This may cause the liver to produce excessive free radicals and lipid peroxidation. Therefore, oxidative stress is one of the main causes of liver fibrosis. Oxidative stress is the molecular mechanism of underlying hepatotoxicity 23. Oxidative stress causes hepatocyte death, non-alcoholic steatohepatitis, hepatic fibrosis, and damage to mitochondrial structure and function 65-67. Hepatic TG levels and excessive oxidative stress may cause tissue damage and activate repair mechanisms. Immune cell recruitment, angiogenesis, HSC activation, and subsequent ECM deposition are components of these repair mechanisms. These results clearly indicate that DPx has good potential to inhibit all hepatic damage markers in TAA-induced liver fibrosis 56, 57. DPx has been assumed to stabilize the structural and functional integrity of the hepatocytes membranes and protect against cellular damage induced by TAA and its metabolites.

Oxidative stress increases liver fibrosis which has been implicated in hepatic progression of glycoxidation and lipid peroxidation. Many published reports have revealed that the major causes of liver fibrogenesis is oxidative damage. Oxidative stress is considered a vital pathophysiological mechanism of TAA-induced hepatotoxicity 22-24. Antioxidant enzymes, such as SOD and CAT, and non-enzymatic antioxidants, like GSH, vitamin C, and vitamin E, are used as markers of oxidative stress in liver injury 42. Currently, several antioxidants are being used to treat hepatic injury 68, 69. Similarly, our current study indicated that antioxidant enzymes, such as SOD, CAT, and GSH, were significantly decreased in mice with TAA-induced liver fibrosis. However, during treatment with DPx, all abnormal antioxidant activities of SOD, CAT, and GSH were significantly restored in mice with TAA-induced liver fibrosis mice. In this regard, we can conclude that DPx attenuates liver fibrosis through its antioxidant potential, which enhances the antioxidant defense mechanism present in an organism. MDA is a reactive carbon compound used to identify lipid peroxidation in a sample 69. MDA concentration was dramatically increased in the hepatic tissue of TAA-induced liver fibrosis mice indicating the contribution of ROS-mediated liver damage in the development of fibrosis. The antioxidant and free radical scavenging properties of DPx are hypothesiesed to contribute to its protective effects by lowering the oxidative stress induced by TAA-mediated ROS production. Oxidative stress results not only in the impairment of mitochondrial function and structure but also in hepatic fibrosis and apoptosis 50-52.

Apoptosis is considered a primary mechanism involved in hepatic cell death in hepatic fibrosis 70. After exposure to TAA, hepatic stellate cells enter the area of injury and engulf apoptotic bodies in the dying hepatocytes 43. The key regulators of apoptosis include Bcl-2, Bax, and caspase-3, and alterations in these proteins may result in hepatic mitochondrial damage 70. Various investigators have reported that increased protein expression of p53 induces apoptosis when Bcl-2 homologous 3 is downregulated 71, 72. The data demonstrated that TAA significantly elevated Bax, cleaved caspase-3, and p53 levels associated with a decrease in Bcl-2 and caspase-3 in the mouse liver. Exposure to TAA concomitantly with DPx resulted in the return of all apoptotic proteins expression levels compared to control values. Similarly, Yang et al. (2019) found that an extract of *D. morbifera* also reversed the effects of TAA-induced apoptotic proteins, including that of Bax, Bcl-2, cleaved caspase-,3 and p53 in rat liver. Interestingly, Park et al. (2020) found that isolated active DPx was effective in reducing cisplatin-initiated increase in Bax and p53 protein expression in the kidney. In addition, the cisplatin-induced decrease in renal Bcl-2 protein expression was elevated by treatment with DPx. In this study, we noted that silymarin also produced effects similar to that of DPx on TAA-induced changes in the expression levels of all apoptotic proteins. These results indicate that DPx or Silymarin diminished liver fibrosis through mechanisms involving an anti-apoptotic pathway.

The total collagen content in liver tissue is an indicator of ECM accumulation, and 4-hydroxyproline is used as a biomarker for hepatic fibrosis 71-73. Lee et al. (2019) reported that TAA-induced elevation in hepatic 4-hydroxyproline levels in rat liver is indicative of tissue fibrosis 74. In agreement with these observations, Yang et al. (2019) demonstrated the TAA-mediated increases in hepatic 4-hydroxyproline content in rats, which was confirmed in our study. Notably, they found that *D. morbifera* extract effectively blocked TAA-mediated increase in hepatic 4-hydroxyproline 20. Our findings showed that isolated active DPx also significantly reduced the 4-hydroxyproline content in TAA-induced liver fibrotic rats, as evidenced by western blotting and immuno staining results. Treatment with silymarin, a positive control, produced effects similar to those of DPx. Thus, the evidence thus indicates that isolated active DPx may be considered a potential candidate to regulate liver fibrosis by lowering 4-hydroxyproline levels and reducing the accumulation of ECM.

The production of ECM protein is directly correlated with the activation of hepatic stellate cells (HSCs). In hepatic fibrosis, accumulation of intracellular fat droplets and differentiation into myofibroblast-like cells occurs in HSC associated with the stimulated upregulated expression of ECM proteins, including collagen-I, α-SMA, TGF-β, α-tubulin, and vimentin 75. The ECM protein expression of α-SMA, collagen-1, TGF-β, α-tubulin, and vimentin was significantly higher in TAA-induced liver fibrosis tissues of rats 19, 20, 76. Our results demonstrated that treatment with isolated active DPx produced inhibitory effects on all the biomarkers related to ECM. This is evidenced by various molecular techniques, such as western blotting, immunostaining and MT- staining. Notably treatment with silymarin, the positive control induced similar effects as did DPx on ECM-related parameters. An earlier report revealed that blocking TGF- β1 signaling pathway decreases liver fibrosis. We observed that mice treated with TAA had significantly higher levels of TGF-β1 expression in the liver. However, TGF-β1 expression was significantly reduced, following DPx treatment. The phosphorylation of Smad2/3 is responsible for the development of liver fibrosis, and its phosphorylation is activated by TGF-β1. Therefore, TGF-β1 is a crucial factor in the study of liver fibrosis as it modulates various associated cellular responses 77, 78. Our current result indicates that administration of DPx dramatically reduced the expression of p-Smad2/3 in mice receiving TAA. However, the MAPK signaling pathway is activated by TGF-β independently, without the involvement of Smad 79.

Our results showed that p-Akt protein expression was significantly higher in TAA-treated mice than in normal mice. The higher expression of p-Akt in TAA injected livers was significantly restored by administration with DPx. Hence, our results suggest that DPx prevents EMT through the TGF-β or non-Smad signaling pathway and that TGF-β1 regulates liver fibrosis via the Akt pathway. However, further clinical studies are required to clarify the mechanism by which DPx decreases the TGF-β1 concentration in the serum of patients with hepatic fibrosis. The proliferation and activation of HSCs, as well as the enhancement of hepatic fibrosis, are caused by members of the MAPK family, namely the main subgroup p38. Therefore, blocking p38 reverses Bax downregulation and Bcl-2 upregulation in activated HSCs 17, 79, 80. The current study revealed that DPx downregulated the expression of p-p38, which was directly correlated with hepatic stellate cell activation and fibrosis induction. Thus, the current study provides first-hand clue *in vivo* evidence that DPx ameliorates hepatic fibrosis by inhibiting TGF-β1/Smad3 and MAPK signaling pathways in hepatocytes of mice administered TAA to induce liver fibrosis.

HSC activation and PI3K/Akt pathway are negatively regulated by PTEN in a phosphatase-dependent manner. PTEN deficiencies have been shown to promote NASH 81. This indicates that an increased PTEN level might prevent HSC activation and growth during hepatic fibrosis. Our data showed that DPx increased PTEN levels, preventing HSC activation and proliferation. An increase in PTEN level is also associated with the activation of the proteolytic cell apoptosis cascade because the inhibition of Akt phosphorylation inhibits HSCs activation and induces apoptosis 82. The Akt signaling pathway may control the proliferative activity of activated HSCs and activate its downstream apoptosis signal, which would causes cellular apoptosis in activated HSCs and reduce fibrogenesis 83. Through the disruption of the p-Akt/Akt pathway, DPx induced HSC apoptosis exerted a protective effect against hepatic fibrosis. The secreted growth factors in stimulated HSCs can activate the p38, which also functions as a significant signaling pathway 84, 85. DPx-mediated decrease in HSC stimulation resulted in the inhibition of TAA-induced fibrotic effects in the liver. A recent study revealed that, DPx inhibited HSC activation in the HSC LX-2 cell line through autophagy inhibition 86. Therefore, DPx can inhibit HSC activation leading to an attenuation of hepatic fibrosis.

Suppression of IL-1β, IL-6, and TNF-α ameliorates hepatic aggravation in fibrogenesis. Hepatic macrophages play a critical role in liver fibrogenesis 87. Furthermore, TNF-related signaling triggers cytokine secretion and immune cell recruitment, which are directly correlated with hepatic inflammation 88. According to this study, DPx potently inhibited pro-inflammatory cytokines (IL-1, IL-6, and TNF-α), which may be key factors in preventing hepatic fibrosis. NAD is used as a cofactor for NAD+ deacetylase in SIRT1 and SIRT3 proteins to deacetylate cellular proteins. Acetylation of all proteins in the mitochondria plays a crucial role in the regulation of proteins and cellular metabolism after acetylating all proteins in the mitochondria 89-93. Different diseases such as metabolic syndrome, cancer, aging, and NAFLD are caused by abnormal lysine residue acetylation 94-97.

Several published reports have revealed that all sirtuin analogues are significantly upregulated in healthy liver 67, 98, 99. Clinically, SIRT1 is significantly downregulated in hepatic disorders compared with that in normal patients 100. SIRT3 plays a potential role in regulating mitochondrial function and caloric restriction in metabolic disorders 101. SIRT3, which regulates ATP generation, plays a crucial role in regulating the acetylation levels of the mitochondrial electron transport chain 102. Mice lacking SIRT3 have 50% lower levels of ATP in hepatocytes 103. Enzymatic SIRT4 metabolism has played an advantageous role in the development of therapeutic agents for metabolic disorders 99. In the present study, DPx induced upregulations of SIRT1 and SIRT3 by DPx showed amelioration of TAA-induced liver fibrosis. However, SIRT 4 revealed the reverse effect in the liver of fibrosis mice. Hence, effective, potentially secure treatment is in high demand for the treatment of liver fibrosis and cirrhosis. Our results proposed that the upregulation of SIRT1, SIRT3 and downregulation of SIRT-4 is a promising approach for protecting against liver fibrogenesis. However, further research is required to determine the precise molecular strategies used by members of the SIRT family to combat hepatic fibrosis. Investigating whether each SIRT subtype performs a particular role in hepatic fibrosis would be interesting.

Hepatocyte apoptosis, necrosis and oxidative stress caused by mitochondrial abnormality, membrane permeabilization, and ATP depletion have been associated with hyperlipidemia 104-106. Therefore, preserving the functional and structural integrity of the mitochondrial is currently the primary approach for the management of hepatic fibrosis. Our study showed that all the abnormalities associated with oxidative stress in liver fibrosis were significantly restored by treatment with DPx, which preserved hepatic mitochondrial function impairment by TAA. Additionally, DPx significantly restored the hepatic morphology. The beneficial effect of DPx may be associated with blocking the activation of TGF-β. DPx reduced apoptosis in hepatocytes. Furthermore, we observed that oxidative stress and cytokine markers were significantly restored following treatment with DPx in the liver fibrosis model. It significantly downregulated the expression of fibrous biomarkers in the livers of BALB/_C_ mice model. Therefore, anti-fibrotics and anti-inflammatory effects exerted by DPx may provide a protective effect in hepatic fibrosis. In conclusion, we believe that our findings in hepatic fibrosis disorders provide deep insight into the (patho-) physiological roles of the TGF-β/Smad signaling pathway in hepatic fibrosis.

In conclusion, our results demonstrate that isolated active DPx attenuates the pathophysiological mechanisms involved in hepatic fibrosis induced by TAA in male BALB/_C_ mice. We observed that DPx prevented TAA-induced hepatic fibrosis by inhibiting oxidative stress, exerting anti-inflammatory and mitochondrial protective effects, decreasing TGF-β1 regulation and reducing apoptosis. These data suggest that DPx is a potential liver-protective agent similar to silymarin. Further research is required to determine the efficacy and potential adverse effects of DPx in clinical practice.

## Figures and Tables

**Table 1:** Primer sequence of genes used for RT-PCR analysis

Sirt1	Forward	GAA CCT CTG CCT CAT CTA
	Reverse	TAC TCG CCA CCT AAC CTA
Sirt3	Forward	AAG ACA TAC GGG CTG ACG
	Reverse	ATC TGC CAA GGC GAA AT
Sirt4	Forward	CGC TTC ATT AGC CTT TCC A
	Reverse	GGC GTA GAG TCC CAC CTT T
β-actin	Forward	CGT TGA CAT CCG TAA AGA CC
	Reverse	TAG AGC CAC CAA TCC ACA CA
